# Efficacy and safety of radiotherapy combined with chemoimmunotherapy versus chemoimmunotherapy alone as first-line treatment for metachronous oligorecurrent esophageal squamous cell carcinoma

**DOI:** 10.3389/fonc.2026.1803137

**Published:** 2026-06-17

**Authors:** Xiaoyan Lv, Yuan Wang, Jingyi Ai, Siyu Chen, Yi Tan, Xinbing Li, Xintong Yan, Jun Wang

**Affiliations:** 1 Department of Radiation Oncology, the Fourth Hospital of Hebei Medical University, Hebei Clinical Research Center for Radiation Oncology, Shijiazhuang, China; 2 Hebei Medical University, Shijiazhuang, China

**Keywords:** chemoimmunotherapy, esophageal squamous cell carcinoma, metachronous oligorecurrence, oligometastatic disease, radiotherapy

## Abstract

**Purpose:**

Metachronous oligorecurrence is usually defined as oligometastatic disease diagnosed more than 6 months after primary tumor diagnosis in the absence of active systemic therapy. This study aimed to assess the safety and efficacy of radiotherapy combined with chemoimmunotherapy (RCIT) versus chemoimmunotherapy (CIT) alone as the first-line treatment for metachronous oligorecurrent esophageal squamous cell carcinoma (MOR-ESCC).

**Methods:**

We retrospectively evaluated 195 patients with MOR-ESCC treated with first-line RCIT (n=102) or CIT (n=93) between June 2018 and June 2023. Propensity score matching (PSM) was performed to reduce baseline imbalance and potential confounding.

**Results:**

Following 1:1 PSM, 72 well-paired patients were identified. The median follow-up was 17.9 months. RCIT was associated with significantly longer progression-free survival (PFS) than CIT, while overall survival (OS) showed a borderline non-significant trend favoring RCIT. The median PFS was 13.1 months (95% CI: 8.9-17.3) in the RCIT group and 9.0 months (95% CI: 6.9-11.1) in the CIT group (*P* = 0.002). The median OS was 21.5 months (95% CI: 13.4-29.7) in the RCIT group and 16.0 months (95% CI: 13.2-18.8) in the CIT group (*P* = 0.051). Subgroup analyses of OS suggested greater benefit from RCIT for patients with recurrence interval ≥18 months (*P* = 0.023), lymph node-only recurrence (*P* = 0.020), 1–2 recurrent lesions (*P* = 0.016), or single-organ recurrence (*P* = 0.022). Safety analysis demonstrated comparable rates of grade ≥3 treatment-related adverse events (TRAEs) (48.6% vs. 34.7%; *P* = 0.091) and treatment-related fatal events (8.3% vs. 4.2%; *P* = 0.491) between groups. Notably, RCIT showed a numerically higher incidence of grade ≥3 pneumonitis (16.7% vs. 5.6%; *P* = 0.063).

**Conclusions:**

RCIT was associated with significantly prolonged PFS and numerically longer OS compared with CIT alone in patients with MOR-ESCC. Although no statistically significant increase in grade ≥3 TRAEs was observed, RCIT showed numerically higher toxicity, particularly pneumonitis. This study provides real-world evidence supporting the role of radiotherapy in MOR-ESCC, identifying long recurrence intervals (≥18 months), nodal-only recurrence, and low tumor burden (1–2 lesions/single organ) as key predictors of RCIT efficacy. These findings support RCIT as a potential strategy for carefully selected patients with MOR-ESCC, although the pneumonitis signal warrants cautious patient selection and prospective validation.

## 1 Introduction

Oligometastatic disease (OMD) is an intermediate stage between locoregional and extensive systemic metastasis and is typically defined by fewer than 3–5 metastatic foci (1). This state may represent a clinically meaningful window in which local consolidative treatment can improve disease control and survival outcomes. To better define this entity, the European Society for Radiotherapy and Oncology (ESTRO) and European Organisation for Research and Treatment of Cancer (EORTC) proposed a nine-category classification system for OMD based on timing (synchronous vs. metachronous), disease burden, and treatment history (2). This framework has contributed substantially to the conceptual refinement of oligometastatic disease and to the identification of patients who may benefit from local ablative treatment.

Despite this progress, current clinical evidence still focuses predominantly on synchronous oligometastatic disease, whereas data on the management of metachronous oligorecurrence (MOR) remain limited. MOR is generally defined as the OMD diagnosed at least 6 months after the diagnosis of the primary tumor in the absence of active systemic therapy at the time of recurrence (2). This entity may reflect a biologically more indolent disease course and may therefore be particularly suitable for local treatment. However, it is worth noting that no global consensus exists regarding the definition of OMD in esophageal squamous cell carcinoma (ESCC), nor is there a uniform definition of MOR as a specific oligometastatic subtype in this disease. Clinical data supporting radiotherapy for metachronous oligorecurrent ESCC (MOR-ESCC) are particularly scarce. The European consensus on oligometastatic esophagogastric cancer limits disease extent to one organ with up to three metastases or one extra-regional lymph node station (3). However, this consensus primarily reflects expert experience based on the Western epidemiological context where more aggressive esophageal adenocarcinoma predominates. Given the prevalence of squamous histology in Chinese patients with esophageal cancer (4) and its distinct biological behavior, the Chinese Expert Consensus on Radiotherapy for Oligometastatic Esophageal Cancer defines oligometastasis as up to 5 metastatic lesions within 3 organs (5). Whether this broader definition is more applicable to MOR-ESCC remains to be determined.

In recurrent ESCC, first-line chemoimmunotherapy (CIT) has improved outcomes compared with historical chemotherapy alone, but survival remains limited and treatment resistance remains common (6–9). From a mechanistic perspective, radiotherapy (RT) may enhance the efficacy of CIT by inducing immunogenic cell death, triggering systemic antitumor immunity, and reversing local immunosuppression. However, whether this potential synergy translates into clinically meaningful benefit in MOR-ESCC is still unclear.

The landmark phase II ESO-Shanghai 13 trial demonstrated significantly improved progression-free survival (PFS) and overall survival (OS) with local therapy added to systemic treatment in oligometastatic ESCC (10). Notably, most enrolled patients had metachronous disease (75/104), and radiotherapy was the predominant local modality used in the combined-treatment arm (44/53). Nevertheless, several limitations restrict the direct applicability of those findings to the present clinical question. First, the lack of subgroup analysis specific to MOR patients obscures whether their outcomes drove the overall benefit, leaving a pivotal evidence gap for MOR-directed therapeutic strategies. Second, only patients with controlled primary tumors were enrolled, whereas local recurrence represents an important pattern of failure in clinical practice. Third, the study was conducted across a treatment era transitioning from chemotherapy-based systemic treatment to immunotherapy-based treatment. Although immunotherapy was incorporated as part of systemic therapy mid-trial, the immunotherapy subgroup ultimately demonstrated no OS benefit, possibly due to insufficient statistical power caused by the small sample size (n=43). Finally, the use of heterogeneous local interventions limited the ability to specifically assess the contribution of radiotherapy.

The present study aimed to evaluate the clinical role of radiotherapy in patients with MOR-ESCC within the EORTC/ESTRO oligometastatic framework by comparing the efficacy and safety of RCIT versus CIT alone as first-line treatment. In addition, we sought to explore clinical subgroups that might derive greater benefit from the addition of radiotherapy.

## 2 Materials and methods

### 2.1 Study design and patients

We retrospectively reviewed patients who received RCIT or CIT with oligorecurrent ESCC at our institution between June 2018 and June 2023. The study was approved by the Medical Ethics Committee of The Fourth Hospital of Hebei Medical University (Ethics review number: 2024KS024). The requirement for informed consent of patients was waived by the board due to the retrospective nature of the study. The inclusion criteria were as follows: histologically or cytologically confirmed ESCC; clinically confirmed recurrent ESCC; with up to five measurable recurrent lesions in no more than three organs; no prior systemic therapy before RCIT or CIT (excluding patients who had progressed after 6 months of [neo]adjuvant therapy/definitive [chemo]radiotherapy); no prior immunotherapy; patients with PFS ≤2 months were excluded due to significant selection bias; an Eastern Cooperative Oncology Group (ECOG) performance status of 0-2; no history of other malignancies or autoimmune diseases; and comprehensive, accessible medical records. Tumor staging was based on the TNM system of the American Joint Committee on Cancer (AJCC 8th edition, 2017).

### 2.2 Radiotherapy

A total of 102 patients in the RCIT group underwent 105 courses of radiation therapy. The median prescription dose to the planning target volume (PTV) was 60 Gy (range: 30.6–66 Gy), with a median fraction dose of 2 Gy (range: 1.8–10 Gy) delivered over a median of 30 fractions (range: 7–33 fractions). Among these, 2 patients received stereotactic body radiation therapy (SBRT), while the remaining patients were treated with intensity-modulated radiation therapy (IMRT). Detailed radiotherapy characteristics of the RCIT cohort are provided in **Supplementary Table 1**, including irradiated target categories, current thoracic RT status, extent of irradiated recurrent lesions, prescription dose/fractionation, biologically effective dose (BED), prior thoracic RT history, and potential field overlap or abutment.

For patients with a history of prior thoracic RT, available previous RT records and treatment plans were reviewed when accessible. Potential overlap or abutment between prior and current irradiation fields was assessed according to treatment site, dose distribution, and available dose-volume histogram information.

### 2.3 Immunotherapy

All patients were treated with anti-PD-1 monoclonal antibodies (mAbs), including pembrolizumab, nivolumab, camrelizumab, toripalimab, sintilimab, and tislelizumab. The median number of cycles for anti-PD-1 mAbs was 4 (range, 1-33; interquartile range [IQR], 2-8) for the RCIT group and 4 (range, 1-54; IQR, 2-6) for the CIT group.

### 2.4 Chemotherapy

Chemotherapy regimens, based on cisplatin or paclitaxel, were administered at 3-week intervals. The median number of chemotherapy cycles was 4 (range, 1-7; IQR, 2-5) in the RCIT group and 4 (range, 1-15; IQR, 2-5) in the CIT group.

### 2.5 PD-L1 expression

PD-L1 testing was performed when tumor tissue was available and at the discretion of the treating physicians; it was not required for study inclusion in this retrospective real-world cohort. PD-L1 expression was assessed in 20 of 102 patients (19.6%) of the RCIT group, including 8 cases with a combined positive score (CPS) <1, 10 cases with CPS 1-49, and 2 cases with CPS≥50; while in the CIT group, 19 of 93 patients underwent PD-L1 testing, including 4 cases with CPS<1, 12 cases with CPS 1-49, and 3 cases with CPS≥50.

### 2.6 Follow-up and statistical analysis

Patients underwent evaluations one-month post-treatment and quarterly after that, which included chest and abdominal computed tomography, barium esophagography, head magnetic resonance imaging, and other imaging and hematological tests. Upon symptoms indicative of disease progression, appropriate imaging was conducted. The study’s primary endpoints were PFS and OS, with PFS defined as the time from initial therapy to the first occurrence of disease progression or death and OS defined as the time from initial therapy to censor or death. The safety of the treatment modalities served as a secondary endpoint. Distant parenchymal organ recurrences excluded the esophagus and lymph nodes. If regional recurrence exists, all positive regional lymph nodes will be classified as one lesion. Positive lymph nodes within the same extra-regional lymph node station are also defined as one lesion.

To reduce baseline imbalance and potential confounding between groups, propensity score matching (PSM) was performed using a 1:1 nearest-neighbor approach with a caliper width of 0.02 on the logit of the propensity score. Propensity scores were estimated using clinically relevant variables available in routine practice, including sex, age, body mass index (BMI), previous definitive treatment, recurrence interval, lymph node-only recurrence, number of recurrent lesions, number of recurrent organs, chemotherapy cycles, and immunotherapy cycles. Categorical variable differences were analyzed using the Chi-square test or Fisher’s exact test, and continuous variables with the Mann-Whitney U test. The Kaplan-Meier method estimated OS and PFS, with between-group differences assessed using the stratified log-rank test. Stratified Cox proportional-hazards models evaluated hazard ratios (HRs) for prognostic factors, with *P* < 0.05 denoting statistical significance.

## 3 Results

### 3.1 Patient characteristics

A total of 195 eligible patients were included, comprising 102 in the RCIT cohort and 93 in the CIT cohort. Before PSM, the RCIT group included a higher proportion of patients with previous definitive treatment and lymph node-only recurrence than the CIT group. After PSM, 72 well-matched patients were identified in each group, and no significant between-group differences in baseline characteristics were observed (**Table 1**).

**Table 1:** Baseline clinical characteristics and treatment exposure of patients before and after PSM.

Characteristics	Before PSM			After PSM		
	RCIT (n=102)	CIT (n=93)	P value	RCIT (n=72)	CIT (n=72)	P value
Sex						
Male	67	66	0.429	50	49	0.857
Female	35	27		22	23	
Age years						
<65	51	36	0.113	35	30	0.402
≥65	51	57		37	42	
BMI						
<18.5	21	24	0.388	14	16	0.682
≥18.5	81	69		58	56	
Previous therapy						
Surgery	73	52	<0.001	46	41	0.394
Definitive RT	29	41		26	31	
Recurrence interval (month)						
<18	40	49	0.059	30	34	0.502
≥18	62	44		42	38	
Lymph node- only recurrence						
Yes	45	21	0.002	22	21	0.856
No	57	72		50	51	
No. of recurrent lesions						
1-2	73	68	0.809	48	52	0.469
3-5	29	25		24	20	
No. of recurrent organs						
1	66	65	0.441	44	47	0.604
2-3	36	28		28	25	
Chemotherapy cycle						
<4	50	46	0.951	35	39	0.505
≥4	52	47		37	33	
Immunotherapy cycle						
<4	46	42	0.993	31	33	0.737
≥4	56	51		41	39	

PSM, Propensity score matching; RCIT, radiotherapy combined with chemoimmunotherapy; CIT, chemoimmunotherapy; RT, radiotherapy; BMI, body mass index.

### 3.2 Overall survival and progression-free survival

As of the last follow-up on December 31, 2024, the median follow-up time was 17.9 months (IQR, 8.9-31.7). Four patients were lost to follow-up (three in the RCIT group and one in the CIT group), yielding an overall follow-up rate of 98.1%. At the time of data cutoff, 134 patients had died.

**Figure 1:**
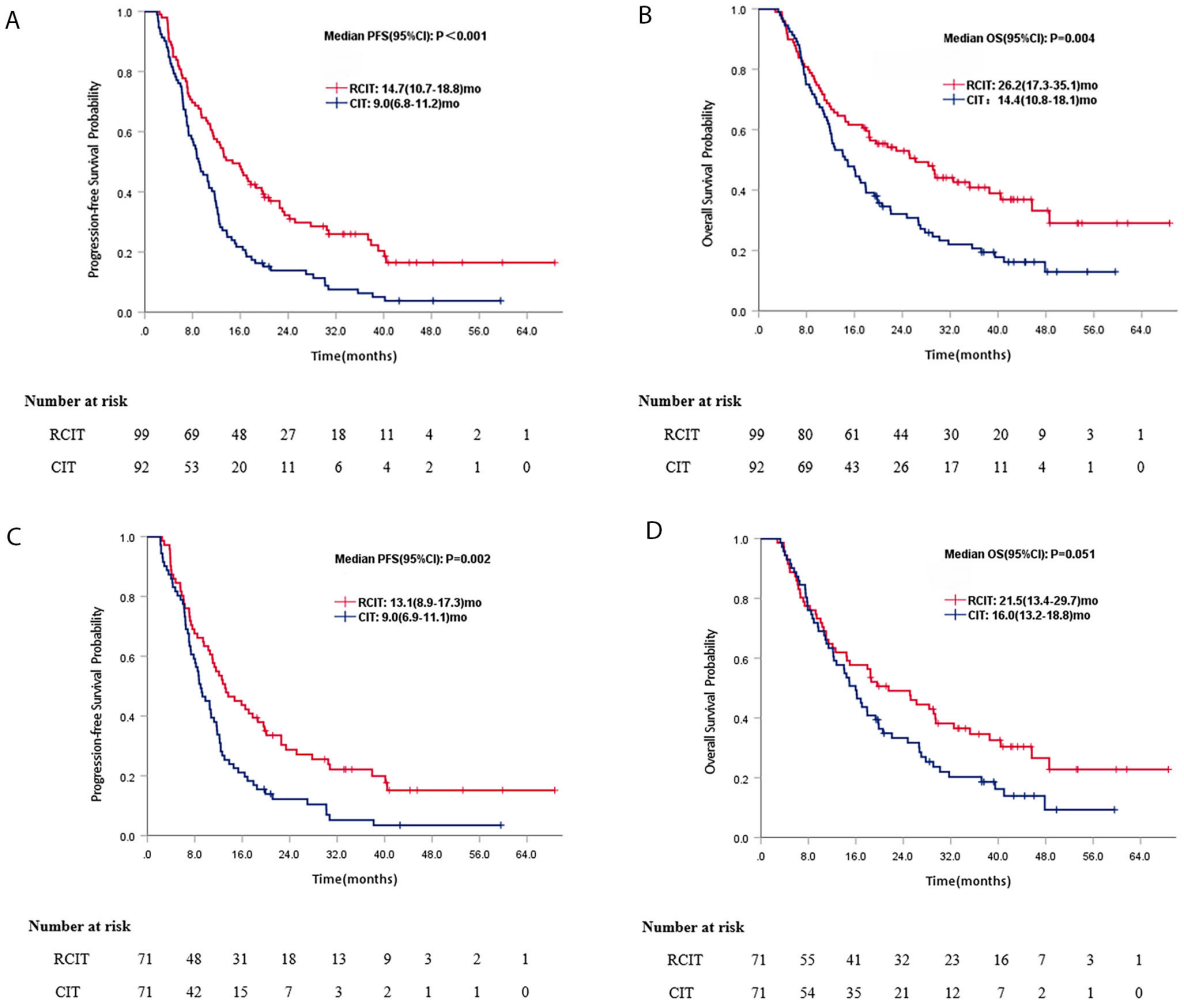
Kaplan-Meier curves of PFS **(A)** and OS **(B)** comparing the RCIT group and the CIT group before PSM, and of PFS **(C)** and OS **(D)** comparing the RCIT group and the CIT group after PSM. PFS, progression-free survival; OS, overall survival; PSM, propensity score matching; RCIT, radiotherapy combined with chemoimmunotherapy; CIT, chemoimmunotherapy.

Prior to PSM, the RCIT group demonstrated significantly superior PFS and OS compared to the CIT group: median PFS (mPFS) was 14.7 months (95% CI: 10.7–18.8) in the RCIT group versus 9.0 months (95% CI: 6.8–11.2) in the CIT group (*P* < 0.001; **Figure 1A**). Median OS (mOS) was 26.2 months (95% CI: 17.3–35.1) in the RCIT group versus 14.4 months (95% CI: 10.8–18.1) in the CIT group (*P* = 0.004; **Figure 1B**). OS rates at 12, 24, and 36 months were 67.7%, 44.4%, and 23.2% in the RCIT group, while corresponding rates in the CIT group were 58.7%, 28.3%, and 17.4%.

**Figure 2:**
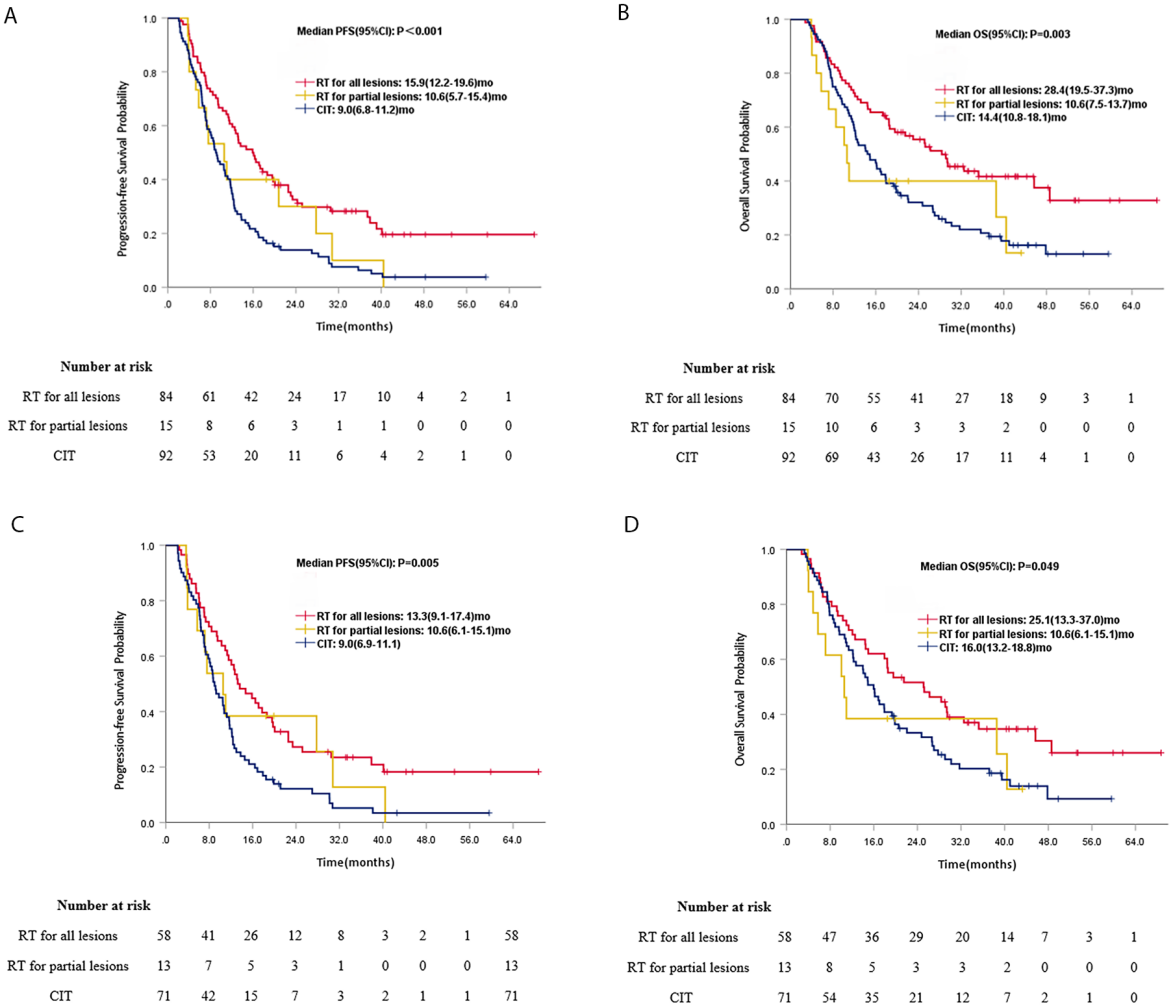
Kaplan-Meier curves of PFS **(A)** and OS **(B)** comparing the complete-lesion RT group, the partial-lesion RT group, and the CIT group before PSM, and of PFS **(C)** and OS **(D)** comparing the complete-lesion RT group, the partial-lesion RT group, and the CIT group after PSM. PFS, progression-free survival; OS, overall survival; RT, radiotherapy; CIT, chemoimmunotherapy; PSM, propensity score matching.

Prior to PSM, significant differences in PFS and OS were also observed among the complete-lesion RT, partial-lesion RT, and CIT alone groups (**Figures 2A, B**). Median PFS was 15.9 months (95% CI: 12.2-19.6), 10.6 months (95% CI: 5.7-15.4), and 9.0 months (95% CI: 6.8-11.2), respectively (P<0.001), whereas mOS was 28.4 months (95% CI: 19.5-37.3), 10.6 months (95% CI: 7.5-13.7), and 14.4 months (95% CI: 10.8-18.1) respectively (*P* = 0.003). Although no statistically significant difference in PFS was observed between complete-lesion RT and partial-lesion RT groups (15.9 vs. 10.6 months, *P* = 0.193), their OS difference reached statistical significance threshold (28.4 vs. 10.6 months, *P* = 0.050). Partial-lesion RT showed no significant survival differences versus CIT in either mPFS (10.6 vs. 9.0 months, *P* = 0.379) or median OS (10.6 vs. 14.4 months, *P* = 0.821), whereas complete-lesion RT demonstrated significant survival advantages over CIT in both mPFS (15.9 vs. 9.0 months, *P* < 0.001) and mOS (28.4 vs. 14.4 months, *P* < 0.001).

After PSM, the RCIT group achieved significantly longer PFS than the CIT group, whereas OS numerically favored RCIT but did not reach conventional statistical significance. The mPFS was 13.1 months (95% CI: 8.9-17.3) for the RCIT group and 9.0 months (95% CI: 6.9-11.1) for the CIT group (*P* = 0.002; **Figure 1C**). Median OS was 21.5 months (95% CI: 13.4-29.7) in the RCIT group and 16.0 months (95% CI: 13.2-18.8) in the CIT group (*P* = 0.051; **Figure 1D**). The 12-, 24-, and 36-month OS rates were 63.9%, 44.4%, and 25.0% in the RCIT group, compared with 62.5%, 29.2%, and 16.7% for the CIT group.

After PSM, significant differences persisted in PFS and OS among patients receiving complete-lesion RT, partial-lesion RT, and CIT alone (**Figures 2C, D**). Median PFS was 13.3 months (95% CI: 9.1–17.4), 10.6 months (95% CI: 6.1–15.1), and 9.0 months (95% CI: 6.9–11.1) respectively (*P* = 0.005), whereas mOS was 25.1 months (95% CI: 13.3–37.0), 10.6 months (95% CI: 6.1–15.1), and 16.0 months (95% CI: 13.2–18.8), respectively (*P* = 0.049). Overall, complete-lesion RT was associated with the most favorable survival outcomes, whereas partial-lesion RT did not demonstrate a clear survival advantage over CIT alone (PFS: 10.6 vs. 9.0 months, *P* = 0.293; OS: 10.6 vs. 16.0 months, *P* = 0.772).

**Figure 3:**
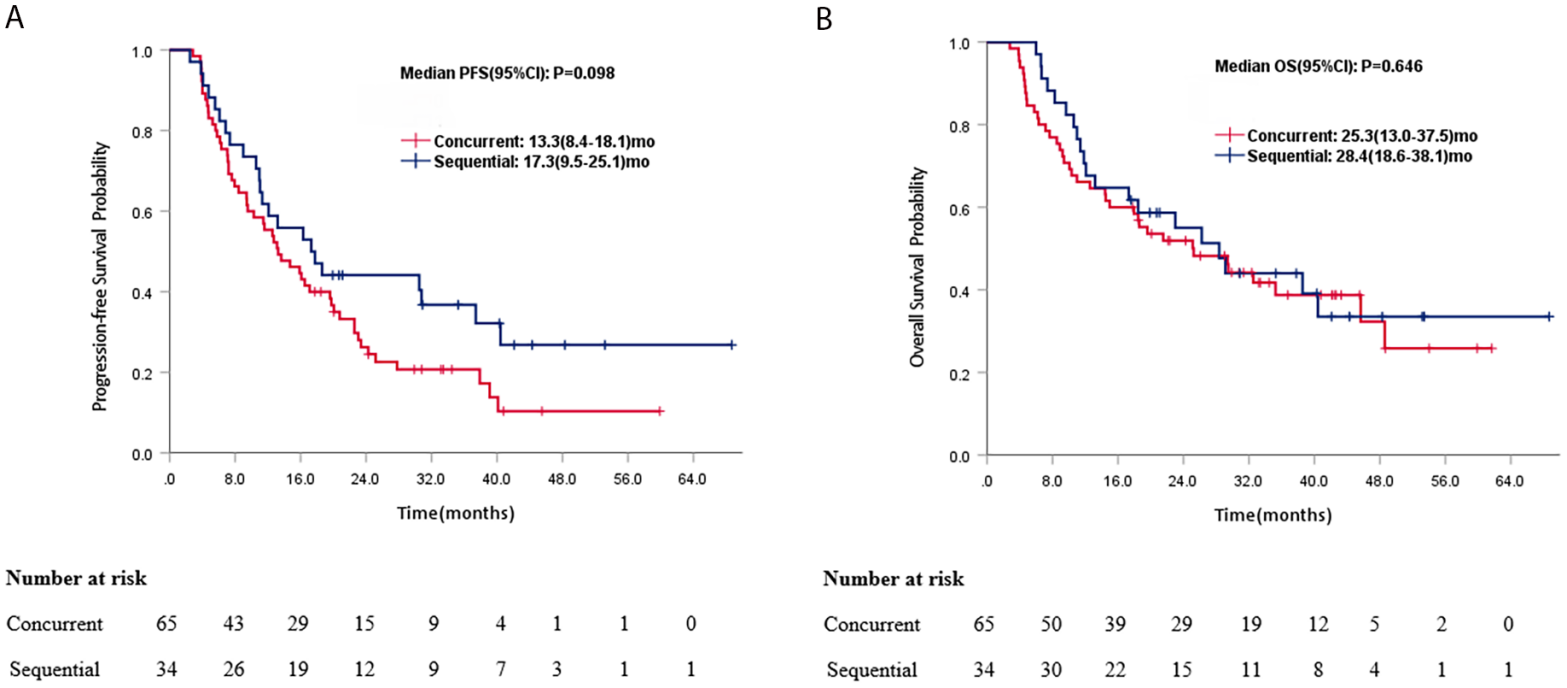
Kaplan-Meier curves of PFS **(A)** and OS **(B)** comparing the concurrent RT group and the sequential RT group. PFS, progression-free survival; OS, overall survival; RT, radiotherapy.

To explore the potential impact of radiotherapy timing on prognosis, we further divided the 102 patients who received RT into a concurrent group (n = 67), defined as receiving RT during immunotherapy, and a sequential group (n = 35), defined as not receiving immunotherapy within 7 days before or after RT. Two patients in the concurrent group and 1 in the sequential group were lost to follow-up. No significant difference in OS was observed between the sequential and concurrent groups (mOS: 28.4 months [95% CI: 18.6–38.1] vs. 25.3 months [95% CI: 13.0–37.5]; *P* = 0.646). However, PFS tended to be longer in the sequential group, although the difference did not reach statistical significance (mPFS: 17.3 months [95% CI: 9.5–25.1] vs. 13.3 months [95% CI: 8.4–18.1]; *P* = 0.098) (**Figure 3**).

The results of univariate and multivariable Cox regression analyses for PFS and OS in the matched cohort are summarized in **Table 2**. In univariate analysis for PFS, recurrence interval (HR = 1.972, 95% CI: 1.381-2.816; *P* < 0.001), lymph node-only recurrence (HR = 0.555, 95% CI: 0.370-0.833; *P* = 0.004), number of recurrent lesions (HR = 0.460, 95% CI: 0.315-0.671; *P* < 0.001), number of recurrent organs (HR = 0.647, 95% CI: 0.450-0.930; *P* = 0.019), chemotherapy cycles (HR = 1.900, 95% CI: 1.274-2.834; *P* = 0.002), immunotherapy cycles (HR = 2.259, 95% CI: 1.511-3.377; *P* < 0.001), and receipt of radiotherapy (HR = 0.563, 95% CI: 0.393-0.807; *P* = 0.002) were associated with PFS. Multivariate analysis for PFS identified recurrence interval (HR = 1.831, 95% CI: 1.241-2.701; *P* = 0.002), number of recurrent lesions (HR = 0.589, 95% CI: 0.356-0.977; *P* = 0.041), immunotherapy cycles (HR = 2.983, 95% CI:1.810-4.915; *P* < 0.001), and receipt of radiotherapy (HR = 0.470, 95% CI: 0.317-0.697; *P* < 0.001) as independent factors affecting PFS. For OS, univariate analysis revealed associations with recurrence interval (HR = 1.980, 95% CI: 1.351-2.901; *P* < 0.001), lymph node-only recurrence (HR = 0.487, 95% CI: 0.311-0.764; *P* = 0.002), number of recurrent lesions (HR = 0.537, 95% CI: 0.362-0.798; *P* = 0.002), number of recurrent organs (HR = 0.545, 95% CI: 0.370-0.803; *P* = 0.002), chemotherapy cycles (HR = 2.223, 95% CI: 1.506-3.280; *P* < 0.001), and immunotherapy cycles (HR = 3.506, 95% CI: 2.356-5.218; *P* < 0.001). Multivariate analysis for OS indicated that recurrence interval (HR = 2.093, 95% CI: 1.385-3.162; *P* < 0.001), lymph node-only recurrence (HR = 0.538, 95% CI: 0.325-0.893; *P* = 0.016), and immunotherapy cycles (HR = 2.933, 95% CI: 1.735-4.957; *P* < 0.001) were independent prognostic factors for OS.

**Table 2:** Univariate and multivariable Cox regression analyses for PFS and OS in the matched cohorts.

Variable	PFS				OS			
	Univariate HR(95%CI)	P	Multivariable HR(95%CI)	P	Univariate HR(95%CI)	P	Multivariable HR(95%CI)	P
Gender								
Male vs. Female	1.112 (0.764-1.616)	0.580	–		1.067 (0.717-1.586)	0.750	–	
Age years								
<65 vs. ≥65	1.316 (0.925-1.874)	0.127	–		1.236 (0.846-1.806)	0.274	–	
BMI								
<18.5 vs. ≥18.5	1.143 (0.746-1.753)	0.540	–		1.205 (0.760-1.912)	0.428	–	
Previous therapies								
Esophagectomy vs. Definitive RT	0.911 (0.635-1.305)	0.610	–		0.783 (0.534-1.150)	0.213	–	
Recurrence interval (month)								
<18 vs. ≥18	1.972 (1.381-2.816)	<0.001	1.831 (1.241-2.701)	0.002	1.980 (1.351-2.901)	<0.001	2.093 (1.385-3.162)	<0.001
Lymph node recurrence only								
Yes vs. No	0.555 (0.370-0.833)	0.004	0.669 (0.421-1.063)	0.089	0.487 (0.311-0.764)	0.002	0.538 (0.325-0.893)	0.016
No. of recurrent lesions								
1–2 vs. 3-5	0.460 (0.315-0.671)	<0.001	0.589 (0.356-0.977)	0.041	0.537 (0.362-0.798)	0.002	0.930 (0.573-1.509)	0.768
No. of recurrent organs								
1 vs. 2-3	0.647 (0.450-0.930)	0.019	1.117 (0.688-1.812)	0.655	0.545 (0.370-0.803)	0.002	0.885 (0.542-1.444)	0.624
Chemotherapy cycle								
<4 vs. ≥4	1.900 (1.274-2.834)	0.002	0.838 (0.513-1.368)	0.480	2.223 (1.506-3.280)	<0.001	1.090 (0.646-1.839)	0.747
Immunotherapy cycle								
<4 vs. ≥4	2.259 (1.511-3.377)	<0.001	2.983 (1.810-4.915)	<0.001	3.506 (2.356-5.218)	<0.001	2.933 (1.735-4.957)	<0.001
Treatment								
RCIT vs. CIT	0.563 (0.393-0.807)	0.002	0.470 (0.317-0.697)	<0.001	0.686 (0.468-1.005)	0.053	0.693 (0.467-1.027)	0.068

Variables with a P-value less than 0.1 in the univariate analysis were included in the multivariate model. BMI, body mass index; RCIT, radiotherapy combined with chemoimmunotherapy; CIT, chemoimmunotherapy; HR, hazard ratio; CI, confidence interval.

### 3.3 Subgroup analysis

**Figure 4:**
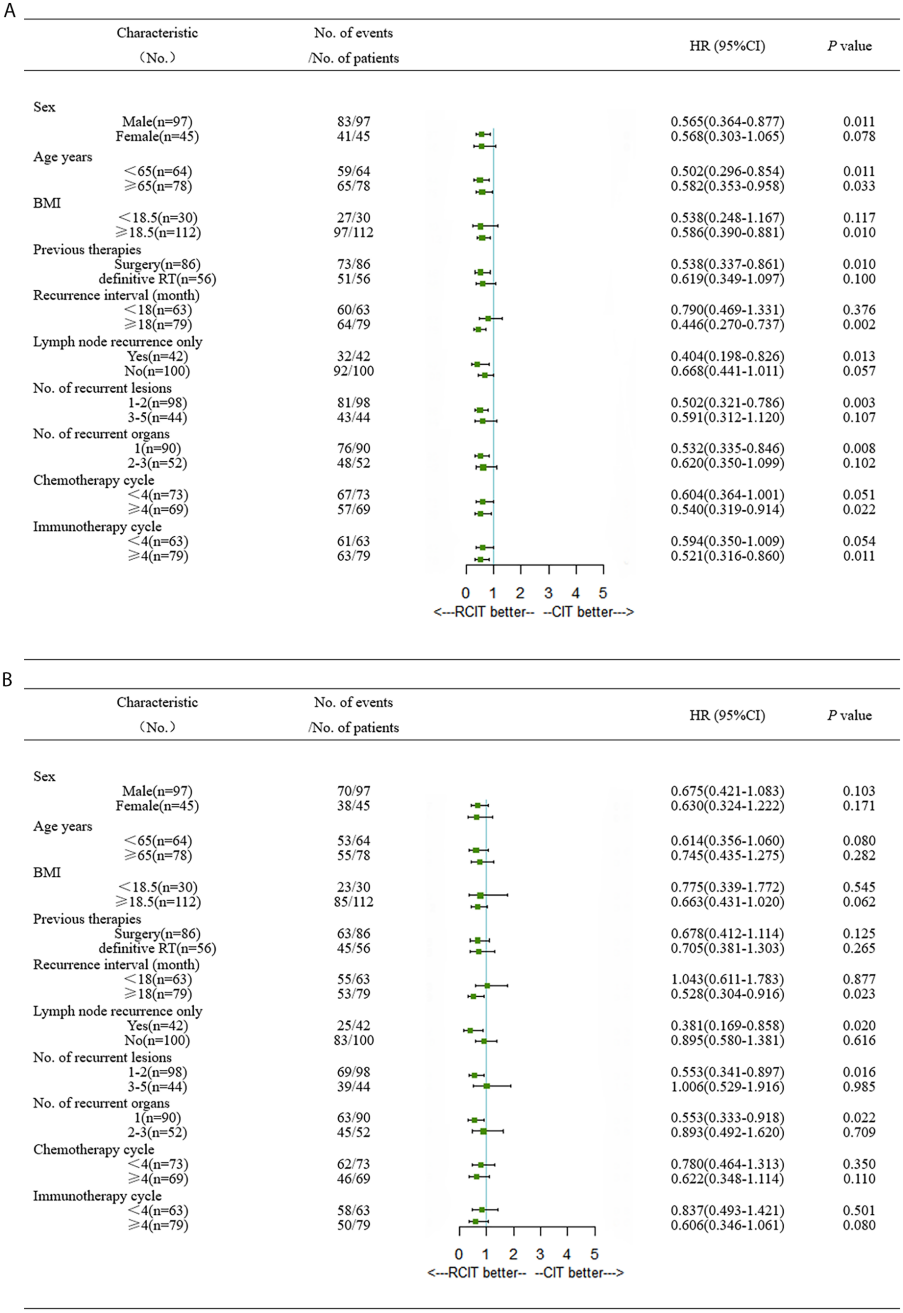
Forest plots of subgroup analyses for PFS **(A)** and OS **(B)** in the propensity score-matched cohorts. PFS, progression-free survival; OS, overall survival.

Subgroup analyses of PFS(**Figure 4A**) demonstrated that compared to CIT, the following patient subgroups derived significantly greater benefit from RCIT: males (HR = 0.565, 95% CI: 0.364–0.877; *P* = 0.011), age <65 years (HR = 0.502, 95% CI: 0.296–0.854; *P* = 0.011) or ≥65 years (HR = 0.582, 95% CI: 0.353–0.958; *P* = 0.033), BMI ≥18.5 kg/m² (HR = 0.586, 95% CI: 0.390–0.881; *P* = 0.010), initial treatment with esophagectomy (HR = 0.538, 95% CI: 0.337–0.861; *P* = 0.010), recurrence interval ≥18 months (HR = 0.446, 95% CI: 0.270–0.737; *P* = 0.002), lymph node-only recurrence (HR = 0.404, 95% CI: 0.198–0.826; *P* = 0.013), 1–2 recurrent lesions (HR = 0.502, 95% CI: 0.321–0.786; *P* = 0.003), single-organ recurrence (HR = 0.532, 95% CI: 0.335–0.846; *P* = 0.008), receipt of ≥4 chemotherapy cycles (HR = 0.540, 95% CI: 0.319–0.914; *P* = 0.022), and receipt of ≥4 immunotherapy cycles (HR = 0.521, 95% CI: 0.316–0.860; *P* = 0.011).

Subgroup analyses of OS (**Figure 4B**) indicated enhanced RCIT benefits for patients with recurrence interval ≥18 months (HR = 0.528, 95% CI: 0.304–0.916; *P* = 0.023), lymph node-only recurrence (HR = 0.381, 95% CI: 0.169–0.858; *P* = 0.020), 1–2 recurrent lesions (HR = 0.553, 95% CI: 0.341–0.897; *P* = 0.016), or single-organ recurrence (HR = 0.553, 95% CI: 0.333–0.918; *P* = 0.022).

### 3.4 Safety

The grade 3 or higher treatment-related adverse events (TRAEs) before and after PSM are summarized in **Table 3**. Before and after PSM, there was no statistically significant difference in the incidence of G3+ TRAEs (Pre-PSM: 42.2% vs. 33.3%, *P* = 0.205; Post-PSM: 48.6% vs. 34.7%, *P* = 0.091) and treatment-related fatal events (Pre-PSM: 6.9% vs. 3.2%, *P* = 0.409; Post-PSM: 8.3% vs. 4.2%, *P* = 0.491) between the RCIT group and the CIT group. It is noteworthy that prior to PSM, the RCIT group exhibited a significantly higher incidence of G3+ pneumonitis compared to the CIT group (13.7% vs. 4.3%, *P* = 0.043). After PSM, the RCIT group also demonstrated a numerically higher incidence trend of G3+ pneumonitis, though not statistically significant (16.7% vs. 5.6%, *P* = 0.063). Among the 7 grade 5 fatal events in the RCIT group, 5 cases were attributed to pneumonitis (all occurring in the concurrent radiotherapy subgroup), including 1 case of esophageal fistula with concurrent pneumonia; the remaining 2 cases resulted from esophageal-tracheal fistula (1 in the concurrent subgroup and 1 in the sequential subgroup). In the CIT group’s 3 grade 5 fatal events, 2 cases were due to pneumonia (including 1 case of esophageal fistula with concurrent pneumonia), and the remaining case was caused by esophageal-tracheal fistula.

**Table 3:** Grade 3 or higher treatment-related adverse events of the RCIT group and CIT group.

Adverse event	Before PSM			After PSM		
	RCIT (n=102)	CIT (n=93)	P	RCIT (n=72)	CIT (n=72)	P
G3+ TRAEs	43 (42.2%)	31 (33.3%)	0.205	35 (48.6%)	25 (34.7%)	0.091
Treatment-related death	7 (6.9%)	3 (3.2%)	0.409	6 (8.3%)	3 (4.2%)	0.491
Radiation esophagitis	6 (5.9%)	–	–	4 (5.6%)	–	–
Esophageal fistula	9 (8.8%)	8 (8.6%)	0.956	7 (9.7%)	7 (9.7%)	1.000
Hematological toxicity	27 (26.5%)	16 (17.2%)	0.119	21 (29.2%)	12 (16.7%)	0.074
Pneumonitis	14 (13.7%)	4 (4.3%)	0.043	12 (16.7%)	4 (5.6%)	0.063
Cardiotoxicity	2 (2.0%)	2 (2.2%)	1.000	2 (2.8%)	1 (1.4%)	1.000
Abnormal liver function	1 (1.0%)	4 (4.3%)	0.312	1 (1.4%)	4 (5.6%)	0.363
Hypophysitis	1 (1.0%)	1 (1.1%)	1.000	0 (0.0%)	0 (0.0%)	–
Abnormal renal function	0 (0.0%)	1 (1.1%)	0.477	0 (0.0%)	1 (1.4%)	1.000
Myositis	0 (0.0%)	1 (1.1%)	0.477	0 (0.0%)	1 (1.4%)	1.000
Peripheral neuritis	1 (1.0%)	0 (0.0%)	1.000	1 (1.4%)	0 (0.0%)	1.000
Autoimmune hemolytic anemia	0 (0.0%)	1 (1.1%)	0.477	0 (0.0%)	0 (0.0%)	–
Hyperglycemia	1 (1.0%)	0 (0.0%)	1.000	0 (0.0%)	0 (0.0%)	–
Rash	1 (1.0%)	0 (0.0%)	1.000	1 (1.4%)	0 (0.0%)	1.000

RCIT, radiotherapy combined with chemoimmunotherapy; CIT, chemoimmunotherapy; G3+, grade 3 or higher; TRAEs, treatment-related adverse events.

The incidence of esophageal fistula was 9.0% (6 of 67) in the concurrent radiotherapy group and 8.6% (3 of 35) in the sequential group (*P* = 1.000). The concurrent group showed a trend toward higher incidence of G3+ pneumonitis compared to the sequential group (17.9% [12 of 67] vs. 5.7% [2 of 35], *P* = 0.163). Given the observed pneumonitis signal, we further reviewed available prior RT records and current RT plans for patients who developed grade ≥3 pneumonitis in the RCIT group. Detailed information on pneumonitis grade, RT-ICI sequencing, current thoracic RT status, current RT dose/fractionation, GTV/CTV/PTV volumes, current lung dose parameters, prior thoracic RT history, re-irradiation interval, field overlap or abutment, and concomitant fistula is summarized in **Supplementary Table 2**. Among the 14 patients with grade ≥3 pneumonitis in the RCIT group, 12 received concurrent RT and 2 received sequential RT. All pneumonitis-related fatal events in the RCIT group occurred in patients who received concurrent RT.

## 4 Discussion

The mOS of patients with metastatic or recurrent ESCC receiving paclitaxel or 5-fluorouracil combined with platinum remains limited to 7–13 months (11–13). Contemporary CIT has extended mOS to 12.6-17.2 months and mPFS to 5.8-7.3 months in the first-line setting (6–9). However, the low response rate, high locoregional failure rate, and acquired resistance to CIT indicate substantial unmet therapeutic needs. Notably, oligometastatic recurrence after radical treatment correlates with improved survival in recurrent esophageal cancer (14). In parallel, emerging evidence has supported the safety and efficacy of combining radiotherapy with systemic chemotherapy in oligometastatic ESCC, with mOS reaching 24.6 months in selected patients (15–17). Nevertheless, robust evidence specifically addressing the role of radiotherapy combined with chemoimmunotherapy in MOR-ESCC remains limited. In this context, the present study provides real-world evidence supporting the clinical applicability of radiotherapy in patients with MOR-ESCC classified according to the EORTC/ESTRO nine-category framework. By comparing RCIT with CIT alone as first-line treatment, our study further suggests that the addition of radiotherapy may improve disease control in this clinically important subgroup. However, given the retrospective and non-randomized design, these findings should still be interpreted cautiously.

In this study, oligometastatic disease was defined according to the Chinese Expert Consensus on Radiotherapy for Oligometastatic Esophageal Cancer, which encompasses a broader scope than the European consensus. While the two consensus definitions differ, neither further stratifies oligometastatic states into synchronous versus metachronous disease. Importantly, the European consensus definition limiting metastases to one extra-regional lymph node station applies only to synchronous oligometastasis and cannot guide the management of metachronous oligometastasis. In our cohort, lesion counting for metachronous oligorecurrence was informed in part by the ESO-Shanghai 13 (10), reflecting a pragmatic approach to patient selection for this clinical scenario. After PSM, the RCIT group demonstrated significantly prolonged PFS and numerically longer OS compared with CIT alone. These findings support the therapeutic relevance of radiotherapy in MOR-ESCC and, to some extent, validate the clinical applicability of radiotherapy for patients categorized within the EORTC/ESTRO oligometastatic framework in a real-world setting. Within such a framework, our findings should not be overinterpreted as redefining oligometastatic disease itself. Instead, they suggest that the benefit of radiotherapy may vary according to recurrence interval, recurrence pattern, and metastatic burden. In particular, patients with recurrence intervals ≥18 months, lymph node-only recurrence, 1–2 recurrent lesions, or single-organ recurrence appeared more likely to benefit from RCIT. These observations indicate that both tumor biology, as reflected by a longer disease-free interval, and disease burden, as reflected by more limited recurrence, may influence the benefit derived from local treatment in MOR-ESCC. Lymph node-only oligorecurrence may represent a clinically favorable subgroup, although this hypothesis requires confirmation in future studies.

Regarding safety, although the incidence of G3+ overall TRAEs showed no significant difference between groups, the RCIT group showed a numerically higher incidence of grade ≥3 pneumonitis, particularly among patients treated with concurrent RT. This toxicity signal is clinically relevant and should be interpreted cautiously. Additional review of radiation plans suggested that severe pneumonitis occurred predominantly in patients receiving concurrent RT, supporting the possibility that concurrent RT during immune checkpoint inhibition, particularly when thoracic or mediastinal targets are covered, may increase the risk of clinically significant pneumonitis in selected patients. Therefore, when RT is integrated with chemoimmunotherapy, especially for thoracic or mediastinal targets, individualized plan review, strict lung dose constraints, and close monitoring during and after treatment are essential. Sequential rather than concurrent RT may be considered for patients with impaired pulmonary reserve, extensive thoracic target volumes, prior thoracic irradiation, or potential field overlap.

Esophageal fistula, a severe complication of advanced esophageal cancer, is associated with a median OS of only 2–8 months after occurrence (18, 19). In this study, esophageal fistula and secondary infectious pneumonitis accounted for 50% of treatment-related mortality events, reaffirming its clinical significance as a poor prognostic indicator. Notably, although high-grade pneumonitis appeared more frequent in the RCIT group, the incidence of esophageal fistula showed no significant difference. Among patients with MOR-ESCC, chemoimmunotherapy still carried a considerable esophageal fistula rate (8.6%), attributable to either rapid tumor regression or progression. Taken together, these findings suggest that when implemented alongside strict patient selection and precision radiotherapy techniques, chemoimmunotherapy combined with radiotherapy may not additionally increase esophageal fistula risk in MOR-ESCC.

Another clinically relevant issue is prior thoracic irradiation. In this real-world cohort, patients with prior thoracic RT were included, and this factor was balanced between groups after PSM. However, formal cumulative OAR dose reconstruction was not performed because complete prior RT DICOM datasets were not consistently available, particularly for patients treated at outside institutions. Therefore, we could not quantitatively assess the contribution of prior irradiation or overlapping/abutting fields to severe pulmonary or esophageal toxicities. For patients requiring re-irradiation or irradiation adjacent to prior fields, cumulative lung, esophageal, tracheobronchial, and cardiac doses should be carefully reviewed. The possibility that prior irradiation or overlapping/abutting fields contributed to severe pneumonitis or fistula in selected patients cannot be excluded.

Several limitations of this study warrant objective consideration. First, this was a retrospective single-institution analysis, and residual confounding may remain despite the use of PSM. Second, PD-L1 expression data were available for only a minority of patients, precluding biomarker-stratified analyses and limiting the interpretability of treatment selection and efficacy evaluation. At the same time, we would like to clarify the real-world treatment context of this study. According to NCCN guidance, PD-L1 testing is recommended in advanced/metastatic esophageal cancer. However, first-line treatment recommendations for esophageal squamous cell carcinoma are regimen-specific rather than uniformly restricted by PD-L1 status. In addition, the anti-PD-1 agents used in our study included several domestically developed PD-1 antibodies commonly used in China. Publicly available prescribing information shows that several domestically developed PD-1 antibodies used in China, including sintilimab, toripalimab, camrelizumab, and tislelizumab, have approved first-line indications for unresectable locally advanced, recurrent, or metastatic esophageal squamous cell carcinoma in combination with chemotherapy, without PD-L1 positivity being stated as a labeled prerequisite. Therefore, the incomplete PD-L1 testing rate in our cohort should be interpreted as a limitation of retrospective real-world practice and tissue availability, rather than as evidence that treatment was delivered outside routine clinical standards. Third, because of the limited sample size, this study was unable to determine the optimal radiotherapy dose, fractionation, or sequencing strategy. Fourth, although our findings support the clinical applicability of radiotherapy within the EORTC/ESTRO framework in MOR-ESCC, they should not be interpreted as establishing a definitive treatment standard. Finally, owing to the retrospective nature of the study, quality-of-life outcomes, including dysphagia relief and pain control, were not consistently available, even though these endpoints are clinically meaningful when assessing the value of radiotherapy beyond survival. These limitations should be addressed in future prospective studies.

In conclusion, this study provides real-world evidence supporting the clinical applicability of radiotherapy for MOR-ESCC within the EORTC/ESTRO nine-category oligometastatic framework. RCIT was associated with improved disease control, and patients with a longer recurrence interval, lymph node-only recurrence, and lower recurrent burden appeared more likely to derive benefit. These findings support RCIT as a promising treatment strategy for selected patients with MOR-ESCC, while prospective validation remains necessary.

## Data Availability

The original contributions presented in the study are included in the article/**Supplementary Material**. Further inquiries can be directed to the corresponding author.

## References

[1] Hellman S Weichselbaum RR . Oligometastases. J Clin Oncol. (1995) 13:8–10. doi: 10.1200/JCO.1995.13.1.8 7799047

[2] Guckenberger M Lievens Y Bouma AB Collette L Dekker A deSouza NM . Characterisation and classification of oligometastatic disease: a European Society for Radiotherapy and Oncology and European Organisation for Research and Treatment of Cancer consensus recommendation. Lancet Oncol. (2020) 21:e18–28. doi: 10.1016/S1470-2045(19)30718-1 31908301

[3] Kroese TE van Laarhoven HWM Schoppman SF Deseyne PRAJ van Cutsem E Haustermans K . Definition, diagnosis and treatment of oligometastatic oesophagogastric cancer: a Delphi consensus study in Europe. Eur J Cancer. (2023) 185:28–39. doi: 10.1016/j.ejca.2023.02.015 36947929

[4] Smyth EC Lagergren J Fitzgerald RC Lordick F Shah MA Lagergren P . Oesophageal cancer. Nat Rev Dis Primers. (2017) 3:17048. doi: 10.1038/nrdp.2017.48 28748917 PMC6168059

[5] Zhou Z Lv X Wang J Hui Z Pang Q . Expert consensus on radiotherapy for oligometastatic esophageal cancer (2025 edition). Radiat Med Prot. (2025) 6:119–31. doi: 10.1016/j.radmp.2025.05.001 38826717

[6] Sun JM Shen L Shah MA Enzinger P Adenis A Doi T . Pembrolizumab plus chemotherapy versus chemotherapy alone for first-line treatment of advanced oesophageal cancer (KEYNOTE-590): a randomised, placebo-controlled, phase 3 study. Lancet. (2021) 398:759–71. doi: 10.1016/S0140-6736(21)01234-4 34454674

[7] Luo H Lu J Bai Y Mao T Wang J Fan Q . Effect of camrelizumab versus placebo added to chemotherapy on survival and progression-free survival in patients with advanced or metastatic esophageal squamous cell carcinoma: the ESCORT-1st randomized clinical trial. JAMA J Am Med Assoc. (2021) 326:916–25. doi: 10.1001/jama.2021.12836 34519801 PMC8441593

[8] Doki Y Ajani JA Kato K Xu J Wyrwicz L Motoyama S . Nivolumab combination therapy in advanced esophageal squamous-cell carcinoma. New Engl J Med. (2022) 386:449–62. doi: 10.1056/NEJMoa2111380 35108470

[9] Xu J Bai Y Xu N Li E Wang B Wang J . Tislelizumab plus chemotherapy as first-line treatment for advanced esophageal squamous cell carcinoma and gastric/gastroesophageal junction adenocarcinoma. Clin Cancer Res. (2020) 26:4542–50. doi: 10.1158/1078-0432.CCR-19-3561 32561664

[10] Liu Q Chen J Lin Y Ye J Shen W Luo H . Systemic therapy with or without local intervention for oligometastatic oesophageal squamous cell carcinoma (ESO-Shanghai 13): an open-label, randomised, phase 2 trial. Lancet Gastroenterol Hepatol. (2024) 9:45–55. doi: 10.1016/S2468-1253(23)00316-3 37980921

[11] Petrasch S Welt A Reinacher A Graeven U König M Schmiegel W . Chemotherapy with cisplatin and paclitaxel in patients with locally advanced, recurrent or metastatic oesophageal cancer. Brit J Cancer. (1998) 78:511–4. doi: 10.1038/bjc.1998.524 9716036 PMC2063082

[12] Zhang X Shen L Li J Li Y Li J Jin M . A phase II trial of paclitaxel and cisplatin in patients with advanced squamous-cell carcinoma of the esophagus. Am J Clin Oncol-Canc. (2008) 31:29–33. doi: 10.1097/COC.0b013e3181131ca9 18376224

[13] Sun S Yu H Wang H Zhang H Wu X Wang J . Phase II study of S-1 plus cisplatin as first-line therapy in patients with Metastatic Esophageal Carcinoma. Oncol Res Treat. (2019) 42:115–22. doi: 10.1159/000495700 30799403

[14] Ohkura Y Shindoh J Ueno M Lizuka T Udagawa H . Clinicopathologic characteristics of oligometastases from esophageal cancer and long-term outcomes of resection. Ann Surg Oncol. (2020) 27:651–9. doi: 10.1245/s10434-019-08175-0 31898096

[15] Liu Q Zhu Z Chen Y Deng J Ai D Liu Q . Phase 2 study of stereotactic body radiation therapy for patients with oligometastatic esophageal squamous cell carcinoma. Int J Radiat Oncol Biol Phys. (2020) 108:707–15. doi: 10.1016/j.ijrobp.2020.05.003 32417405

[16] Chen Y Cheng X Song H Wu AJ Ku GY Lee P . Outcomes of concurrent chemoradiotherapy versus chemotherapyalone for esophageal squamous cell cancer patients presenting with oligometastases. J Thorac Dis. (2019) 11:1536–45. doi: 10.21037/jtd.2019.03.10 31179097 PMC6531702

[17] Shi Z Zhu X Ke S Qiu H Cai G Zhangcai Y . Survival impact of concurrent chemoradiotherapy for elderly patients with synchronous oligometastatic esophageal squamous cell carcinoma: a propensity score matching and landmark analyses. Radiother Oncol. (2021) 164:236–44. doi: 10.1016/j.radonc.2021.09.033 34627936

[18] Chen HY Ma XM Ye M Hou YL Xie HY Bai YR . Esophageal perforation during or after conformal radiotherapy for esophageal carcinoma. J Radiat Res. (2014) 55:940–7. doi: 10.1093/jrr/rru031 24914102 PMC4202289

[19] Guan X Liu C Zhou T Ma Z Zhang C Wang B . Survival and prognostic factors of patients with esophageal fistula in advanced esophageal squamous cell carcinoma. Biosci Rep. (2020) 40(1):BSR20193379. doi: 10.1042/BSR20193379 31894852 PMC6960064

