# Supplementary material for: Efficacy and safety of radiotherapy combined with chemoimmunotherapy versus chemoimmunotherapy alone as first-line treatment for metachronous oligorecurrent esophageal squamous cell carcinoma
Source: Front Oncol. 2026 Jun 17;16:1803137. doi: 10.3389/fonc.2026.1803137 (PMC13284883; doi:10.3389/fonc.2026.1803137)
Supplement: Supplementary file 1 [file DataSheet1.pdf]

Supplementary Materials

Supplementary Table S1. Radiotherapy characteristics in the RCIT cohort

Supplementary Table S2. Radiation plan characteristics of RCIT patients with grade ⩾3 pneumonitis

Supplementary Table S1. Radiotherapy characteristics in the RCIT cohort

Variable	Overall RCIT (n=102)	Concurrent RT (n=67)	Sequential RT (n=35)
No. of RT courses	105	69	36
Prescription dose, median, range	60 Gy (30.6-66 Gy)	56 Gy (45-66 Gy)	60 Gy (30.6-66 Gy)
Fraction dose, median, range	2 Gy (1.8-10 Gy)	2 Gy (1.8-10 Gy)	2 Gy (1.8-3 Gy)
No. of fractions, median, range	30 (7-33)	28 (7-33)	30 (15-33)
BED, median, range	72 Gy (36.1-100.8 Gy)	67.2 Gy (53.1-100.8 Gy)	72 Gy (36.1-79.2 Gy)
Irradiated target categories, n (%)			
Thoracic/locoregional	78 (76.5%)	49 (73.1%)	29 (82.9%)
Extra-regional lymph node	36 (35.3%)	24 (35.8%)	12 (34.3%)
Distant organ	14 (13.7%)	6 (9.0%)	8 (22.9%)
Current thoracic RT, n (%)			
Yes	81 (79.4%)	51 (76.1%)	30 (85.7%)
No	21 (20.6%)	16 (23.9%)	5 (14.3%)
RT lesion coverage, n (%)			
Complete-lesion RT	87 (85.3%)	57 (85.1%)	30 (85.7%)
Partial-lesion RT	15 (14.7%)	10 (14.9%)	5 (14.3%)
Prior thoracic RT, n (%)	30 (29.4%)	21 (31.3%)	9 (25.7%)
Field overlap/abutment among patients with prior thoracic RT, n (%)			
No	7 (23.3%)	6 (28.6%)	1 (11.1%)
Abutting	5 (16.7%)	3 (14.3%)	2 (22.2%)
Overlapping	10 (33.3%)	6 (28.6%)	4 (44.4%)
Unavailable	8 (26.7%)	6 (28.6%)	2 (22.2%)

Except for the number of RT courses, percentages were calculated using the number of patients in each column as the denominator. Percentages for field overlap/abutment were calculated among patients with prior thoracic RT. Target site categories were not mutually exclusive, and percentages

may therefore exceed 100%. Thoracic/locoregional targets included esophageal recurrence and/or regional lymph node recurrence; extra-regional lymph node targets referred to non-regional nodal stations; and distant organ targets referred to non-nodal distant metastatic sites. Thoracic RT was defined as RT with intended target involvement of intrathoracic structures; supraclavicular or cervical nodal irradiation was not classified as thoracic RT solely because of minor incidental apical lung exposure. Among the 30 patients with prior thoracic RT, 29 had received definitive RT and 1 had received neoadjuvant RT. Field overlap/abutment was assessed from available prior RT records and dose distributions; "Unavailable" indicates that prior RT plan or dose-distribution data could not be retrieved, primarily because prior RT was delivered at outside institutions. BED was calculated using an α/β ratio of 10 Gy. Abbreviations: BED, biologically effective dose; RCIT, radiotherapy combined with chemoimmunotherapy; RT, radiotherapy.

Supplementary Table S2. Radiation plan characteristics of RCIT patients with grade ≥3 pneumonitis

Case	Pneumonitis grade	RT-ICI sequencing	Current thoracic RT	PTV dose/ fractionation	GTV, cm^3	CTV, cm^3	PTV, cm^3	Lung V_5, %	Lung V_{20}, %	MLD, cGy	Prior thoracic RT	Re-RT interval, months	Field overlap/ abutment	Concomitant fistula
1	3	Concurrent	No	50.4 Gy/28 f	42.8	71.4	123.6	-	-	-	Yes	10.2	Abutting	No
2	3	Concurrent	Yes	52 Gy/26 f	51.5	112.0	225.6	16.6	5.0	359.2	Yes	14.7	No	No
3	3	Concurrent	Yes	49.8 Gy/26 f	70.0	171.0	382.6	34.3	15.8	870.7	Yes	15.7	Overlapping	No
4	3	Concurrent	Yes	45 Gy/25 f	35.9	115.0	211.6	30.1	10.4	591.6	No	-	-	Yes
5	3	Concurrent	Yes	54 Gy/30 f	37.5	110.7	212.6	43.6	17.1	894.3	No	-	-	Yes
6	3	Concurrent	Yes	60 Gy/30 f	138.7	303.7	436.3	51.0	25.9	1379.4	No	-	-	No
7	3	Concurrent	No	60 Gy/30 f	32.4	70.0	141.2	-	-	-	No	-	-	No
8	3	Sequential	Yes	60 Gy/30 f	72.0	137.5	197.9	28.4	16.4	773.7	No	-	-	Yes
9	4	Sequential	Yes	40 Gy/16 f	14.6	33.5	61.0	18.1	3.6	306.3	Yes	79.5	Unavailable	No
10	5	Concurrent	Yes	48 Gy/30 f	41.4	120.6	194.3	38.5	13.6	738.1	Yes	48.3	Overlapping	No
11	5	Concurrent	No	54 Gy/30 f	25.1	59.7	98.1	-	-	-	Yes	11.7	No	Yes
12	5	Concurrent	No	30.6 Gy/17 f	61.9	147.0	259.8	-	-	-	No	-	-	No
13	5	Concurrent	Yes	45 Gy/25 f	355.6	500.1	654.3	45.3	21.1	1068.0	No	-	-	No
14	5	Concurrent	Yes	50.4 Gy/28 f	79.0	137.3	231.4	37.2	14.5	757.7	No	-	-	No

Field overlap/abutment was assessed from available prior RT records and dose distributions; "Unavailable" indicates that prior RT plan, dose-distribution, or DVH data could not be retrieved, primarily because prior RT was delivered at outside institutions. "-" indicates not applicable. Concomitant fistula refers to clinically documented esophageal fistula or esophago-respiratory fistula before or during the pneumonitis event. Abbreviations: CTV, clinical target volume; GTV, gross tumor volume; ICI, immune checkpoint inhibitor; MLD, mean lung dose; PTV, planning target volume; Re-RT, re-irradiation; RT, radiotherapy.
